# Supplementary material for: Longitudinal Study of Advanced Non-Small Cell Lung Cancer with Initial Durable Clinical Benefit to Immunotherapy: Strategies for Anti-PD-1/PD-L1 Continuation beyond Progression
Source: Cancers (Basel). 2023 Nov 26;15(23):5587. doi: 10.3390/cancers15235587 (PMC10705796; doi:10.3390/cancers15235587)

Cancer model:

- *Virtual tumor sphere of volume « V » and radius « R »= D/2*
- *D = sum of target lesions per RECIST 1.1*
- *Tumor sphere growth: exponential function of time « t »*

$$V = 4\,\pi\,R^3/3$$

$$V_t = V_0 \exp(TG.t)$$

$$TG = 3\log(Dt/D0)/t$$

$$TGR = 100\,(\exp(TG) - 1)$$

Growth rate, in %/month, of the volume of the global virtual tumor sphere

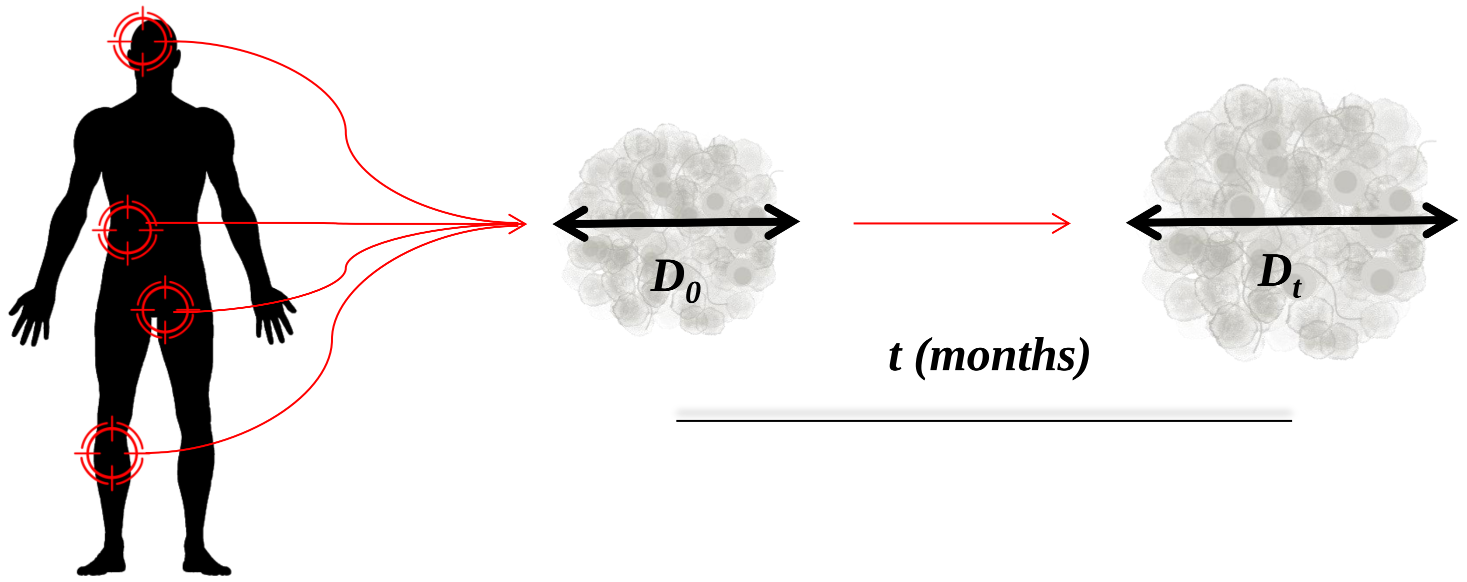

Supplement: Supplementary file 1 [file cancers-15-05587-s001.zip › Supp Fig S1 - TGR.pdf]
