# Supplementary material for: Longitudinal Study of Advanced Non-Small Cell Lung Cancer with Initial Durable Clinical Benefit to Immunotherapy: Strategies for Anti-PD-1/PD-L1 Continuation beyond Progression
Source: Cancers (Basel). 2023 Nov 26;15(23):5587. doi: 10.3390/cancers15235587 (PMC10705796; doi:10.3390/cancers15235587)
Supplement: Supplementary file 1 [file cancers-15-05587-s001.zip › Supp Tables.pdf]

Record ID	TGR at baseline	TGR at PD	(TGR at PD) – (TGR at baseline)
20	0	0	0
34	0	0	0
118	12.388174267142	1.7414546411449683	-10.646719625997
131	12.224674712953787	0	-12.224674712953787
100	159.09177649222505	0	-159.09177649222505
14	20.64531550717712	0	-20.64531550717712
123	24.339482530031532	2.3903980877528763	-21.949084442278654
82	45.495517688939536	22.62471283020291	-22.870804858736626
99	263.9109874292884	-13.598883758907165	-277.5098711881956
143	50.141727065929366	17.897769952690457	-32.243957113238906
139	77.40422359922444	44.235411086081825	-33.16881251314261
96	38.83106847625	0	-38.83106847625
85	53.82943084646092	0	-53.82943084646092
125	543.5177915994036	0	-543.5177915994036
113	3.9356266368534	4.3653138036101	0.42968716675673
51	10.67980381222795	20.852463043851486	10.172659231623536
32	-9.149241654065154	4.358268100050711	13.507509754115866
65	0	20.13360462037759	20.13360462037759
138	11.730493284870281	15.951503891896234	4.221010607025953
130	0	*NA*	*NA*
61	*NA*	20.31635783321264	*NA*
107	*NA*	0	*NA*
135	*NA*	*NA*	*NA*
16	10.93009117412	*NA*	*NA*
95	*NA*	*NA*	*NA*
25	*NA*	27.26758888817349	*NA*
78	*NA*	13.979438856195	*NA*
38	*NA*	-7.0184637492031	*NA*
59	*NA*	0	*NA*
137	*NA*	*NA*	*NA*
89	*NA*	*NA*	*NA*
92	*NA*	8.031584359335241	*NA*
115	*NA*	0	*NA*
116	*NA*	-35.12330378991061	*NA*
56	36.593193844281	*NA*	*NA*
136	*NA*	*NA*	*NA*
142	*NA*	91.11066136359516	*NA*
128	4.174579567361	*NA*	*NA*
127	5.121696112977525	*NA*	*NA*
67	*NA*	*NA*	*NA*
122	*NA*	45.91109068426906	*NA*
129	*NA*	*NA*	*NA*
63	*NA*	*NA*	*NA*
			**(PD – baseline ≤ 0) OR (PD ≤ 0):**<br>n= 19

Supplementary Table S1: Patients with potential benefit of continuing PD-1/PD-L1 blockade beyond PD. Highlighted lines are patients whose TGR assessed at PD was either null/negative, or inferior to TGR measured at baseline before ICI when both time points were available. Total = 19/43 patients.

Oligo PD lesions	New lesions at PD2	Interval PD-PD2 (months)	TGR oligo (%/month)
1	No	2,73	0,00
1	No	2,79	2,93
2	No	3,35	4,80
2	No	2,30	7,33
1	No	1,28	11,53
1	No	3,48	12,12
2	No	2,60	18,93
1	No	3,88	20,07
2	No	1,28	20,32
2	No	2,30	20,85
1	No	1,54	22,24
1	No	3,88	23,00
1	No	1,12	26,12
1	No	2,00	31,16
1	No	1,15	32,12
1	No	2,07	32,71
2	No	2,33	34,20
2	No	2,76	35,04
1	No	2,07	35,15
1	No	1,91	41,68
1	No	3,61	54,62
1	No	2,33	58,30
2	No	1,77	145,31
2	No	2,23	160,37
1	Yes	2,33	*NA*
2	Yes	2,99	*NA*
1	Yes	2,46	*NA*
1	Yes	2,04	*NA*
3	*NA*	*NA*	*NA*
3	*NA*	*NA*	*NA*
1	*NA*	*NA*	*NA*
2	*NA*	*NA*	*NA*
		Mean PD-PD2	Median TGR oligo
		2,378165869	24,56 (IQR 17,84)

Supplementary Table S2: Oligoprogression characterization. « Oligo PD lesions» : number of progressing lesions in OPD under PD-1/PD-L1 blockade ; « New lesions PD2 » : presence of new lesions on « PD2 » imagery following first PD ; « Interval PD-PD2 » : time (months) elapsed between both imageries ; « TGR oligo » : TGR calculated specifically on progressing lesions in assessable OPD patient.

		Primary resistance (N=105)	PD after DCB (N=43)
**No treatment**		38	6
**Subsequent treatment**	Anti-PD-1/PD-L1monotherapy continuation + locoregional treatment	2	3
	Anti-PD-1/PD-L1 monotherapy rechallenge after toxicity	0	3
	Anti-PD-1/PD-L1monotherapy continuation despite PD	9	5
	Locoregional treatment alone	1	2
	New systemic therapy	55	24
	Platine doublet +or- bevacizumab	11	3
	Taxane +or- bevacizumab	25	6
	Tyrosine kinase inhibitor	8	3
	Pemetrexed	5	7
	Vinorelbine	3	0
	Gemcitabine	3	5

Supplementary Table S3: Subsequent treatment after primary resistance or PD after DCB with anti-PD-1/PD-L1.
« Locoregional treatment » : radiation therapy, local ablative therapies or surgery
